# Supplementary material for: A FRET-based respirasome assembly screen identifies spleen tyrosine kinase as a target to improve muscle mitochondrial respiration and exercise performance in mice
Source: Nat Commun. 2023 Jan 25;14:312. doi: 10.1038/s41467-023-35865-x (PMC9877034; doi:10.1038/s41467-023-35865-x)
Supplement: Supplementary file 1 — Supplementary Information [file 41467_2023_35865_MOESM1_ESM.pdf]

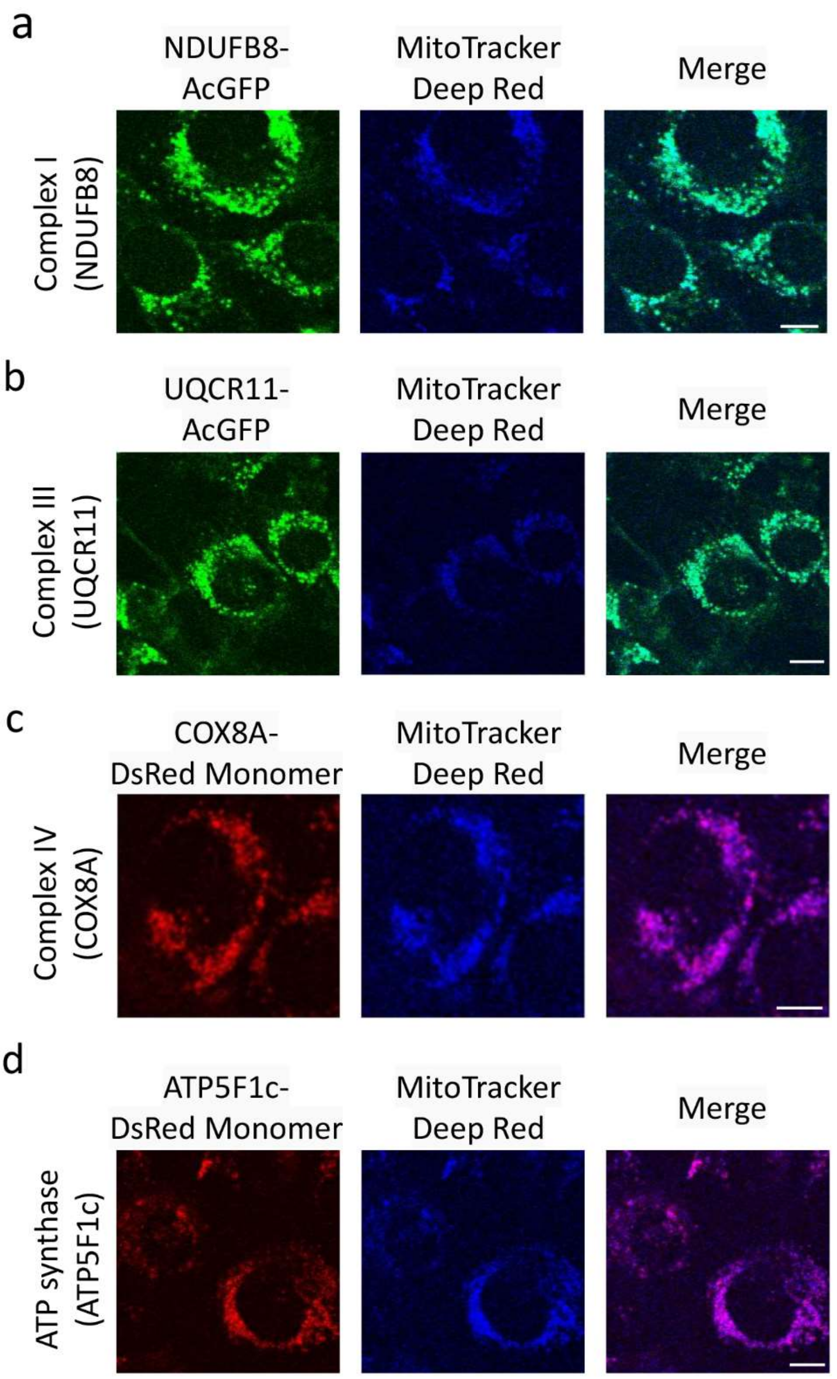

Supplementary Fig. 1. Mitochondrial localization of respiratory chain complex subunits fused with fluorochromes. a-d, Fluorescence microscopy images of C2C12 myoblastic cells expressing (**a**) NDUFB8-AcGFP, (**b**) UQCR11-AcGFP, (**c**) COX8A-DsRed Monomer, and (**d**) ATP5F1c-DsRed Monomer with MitoTracker Deep Red. Fluorescence images of AcGFP channel (**a**, **b**), DsRed-Monomer channel (**c**, **d**), and MitoTracker Deep Red channel, with merged images

are shown. These experiments were repeated twice and the results of one experiment are shown. Scale bars, 10 μm.

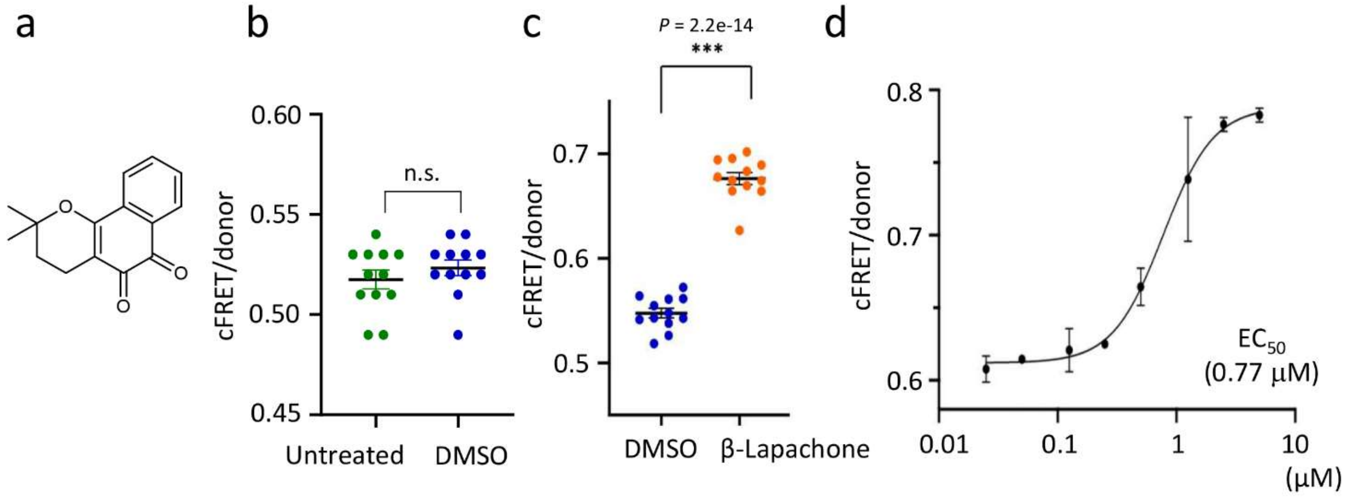

Supplementary Fig. 2. Identification of β-lapachone as a candidate compound promoting mitochondrial respiratory chain supercomplex formation. a, Chemical structure of β-lapachone. **b,** cFRET/donor ratio of C2C12 myoblastic cells stably co-expressing FRET pair of NDUFB8-AcGFP and COX8A-DsRed-Monomer treated with DMSO or untreated. Data from 12 wells are presented as means ± SE. n.s., not significant; unpaired two-sided Student's *t*-test. **c,** cFRET/donor ratio of C2C12 myoblastic cells stably co-expressing FRET pair of NDUFB8-AcGFP and COX8A-DsRed-Monomer treated with vehicle (DMSO) and β-lapachone (1 μM). Data from 12 wells are presented as means ± SE. $^{***}P < 0.001$; unpaired two-sided Student's *t*-test. **d,** cFRET/donor ratio of C2C12 myoblastic cells stably co-expressing FRET pair of NDUFB8-AcGFP and COX8A-DsRed-Monomer treated by different concentrations of β-lapachone. Data from 3 wells in each treatment condition are presented as means ± SE. EC_{50}, half maximal effective concentration. Source data are provided as a Source Data file.

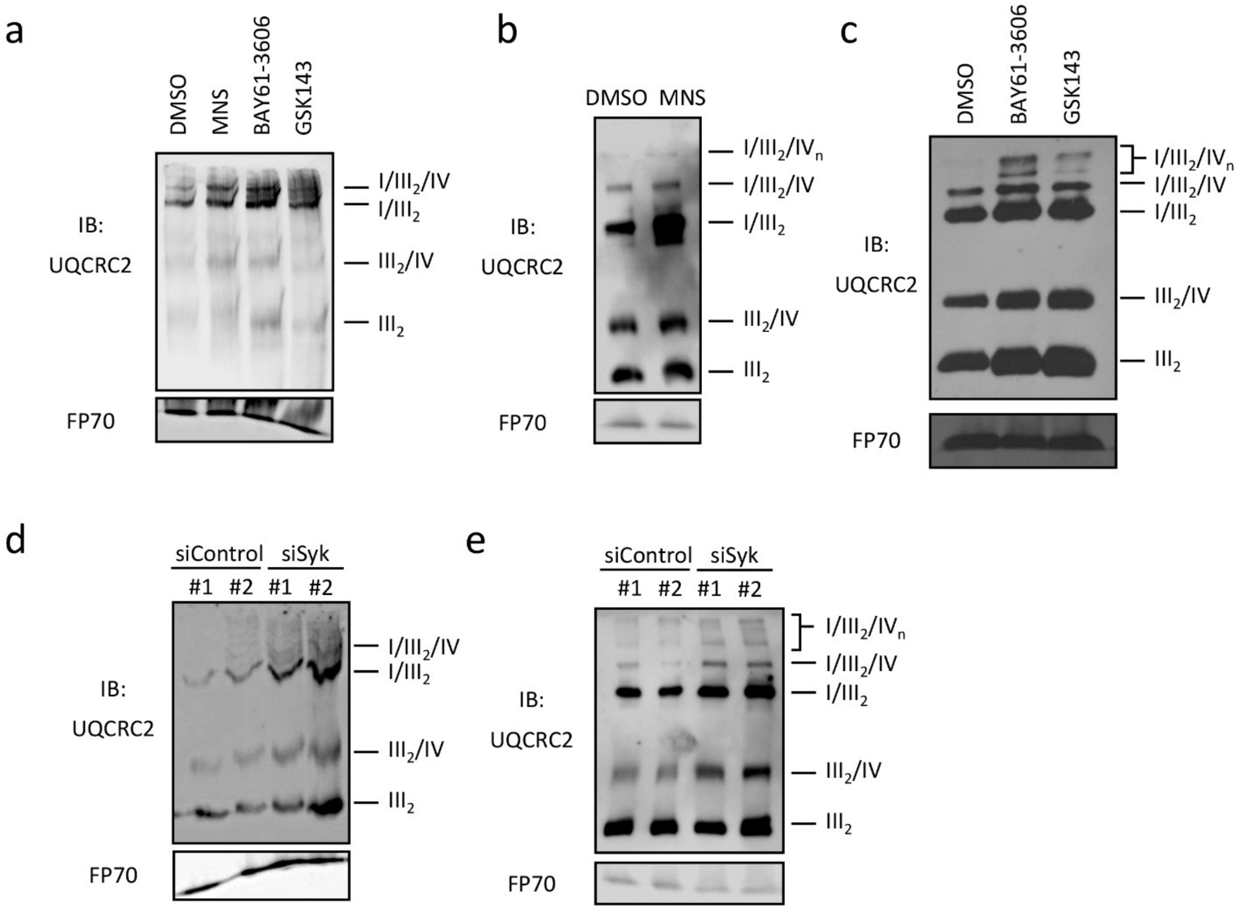

Supplementary Fig. 3. Supercomplex formation in C2C12 cells was enhanced by treatment of SYK inhibitors and siSyk. a-c, Supercomplex formation in C2C12 myoblastic cells treated with MNS (1 μM), BAY61-3606 (1 μM), GSK143 (1 μM), or DMSO for 24 h was analyzed by BN-PAGE. **d, e,** Supercomplex formation in C2C12 myoblastic cells treated with indicated siRNAs (100 pM each) was analyzed by BN-PAGE. Source data are provided as a Source Data file.

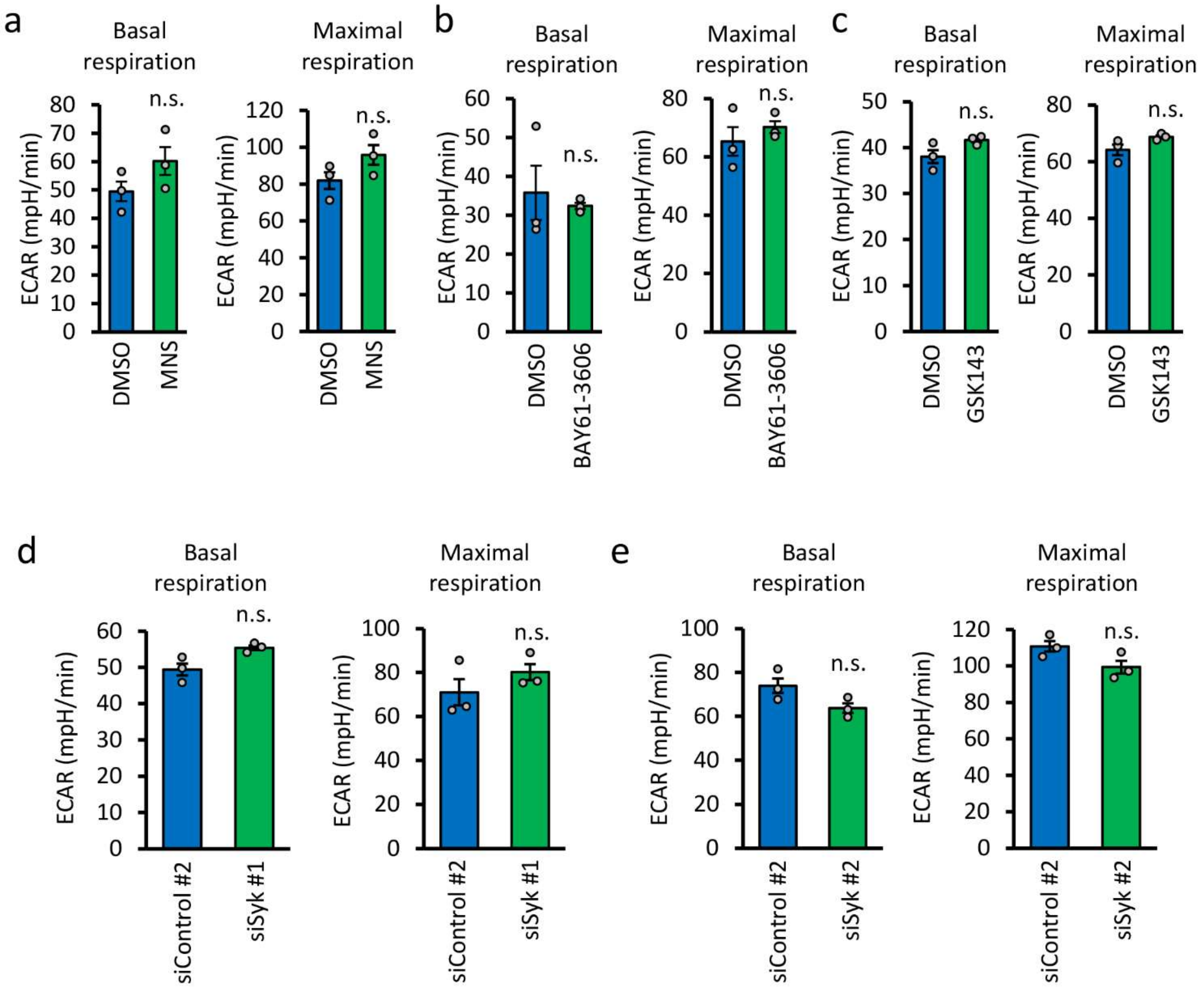

Supplementary Fig. 4. ECARs in C2C12 myoblastic cells were not significantly changed by the treatment of SYK inhibitors and siSyk. a-c, Extracellular acidification rates (ECARs) in C2C12 myoblastic cells treated with MNS (1 μM), BAY61-3606 (1 μM), GSK143 (1 μM), or DMSO for 24 h were measured using Seahorse XFp Cell Mito Stress Test. Data for the timepoint prior to the injection of oligomycin and rotenone/antimycin A (basal and maximal respiration, respectively) are presented as means ± SE ($n = 3$ biologically independent samples). n.s., not significant; unpaired two-sided Student's *t*-test. **d, e,** ECARs in C2C12 myoblastic cells treated with indicated siRNAs for 48 h were measured using Seahorse XFp Cell Mito Stress Test. Data for the timepoint prior to the injection of oligomycin and rotenone/antimycin A (basal and maximal respiration, respectively) are presented as means ± SE ($n = 3$ biologically independent samples). n.s., not significant; unpaired two-sided Student's *t*-test. Source data are provided as a Source Data file.

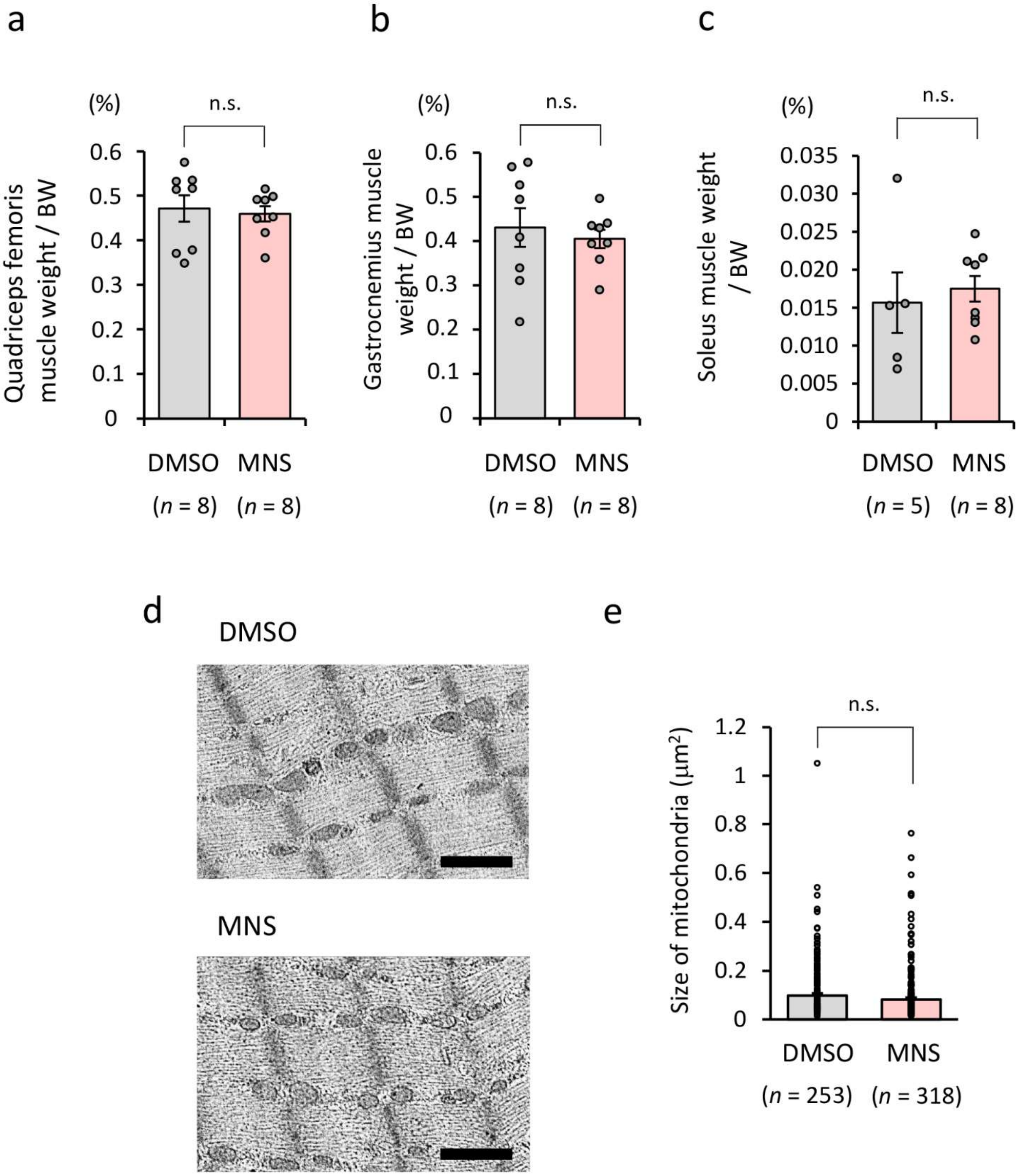

Supplementary Fig. 5. MNS treatment did not affect muscle weight. a-c, Muscle weights of the mice after intraperitoneal injection of MNS (4 mg/kg) or DMSO twice a week for 5 weeks were evaluated. Average weights of quadriceps femoris muscles (**a**), gastrocnemius muscles (**b**), or soleus muscles (**c**) from both right and left legs are divided by body weights. Data are presented

as means ± SE. n.s., not significant; unpaired two-sided Student's *t*-test. **d**, Representative transmission electron micrographs of soleus muscle from the mouse after intraperitoneal injection of MNS or DMSO for 5 weeks. Scale bars; 1 μm. **e**, The size of the mitochondria in the soleus muscle from MNS- or DMSO-treated mice were quantified by transmission electron microscopic examination. Indicated number of mitochondria was evaluated. Sections of soleus muscles from two mice treated with DMSO and two mice treated with MNS were used for quantification of mitochondrial size. For DMSO-treated mice, five sections were prepared from the first mouse and three sections from the second mouse. For the MNS-treated mice, three sections were prepared from each of two mice. Data are presented as means ± SE. n.s., not significant; two-sided median test. Source data are provided as a Source Data file.

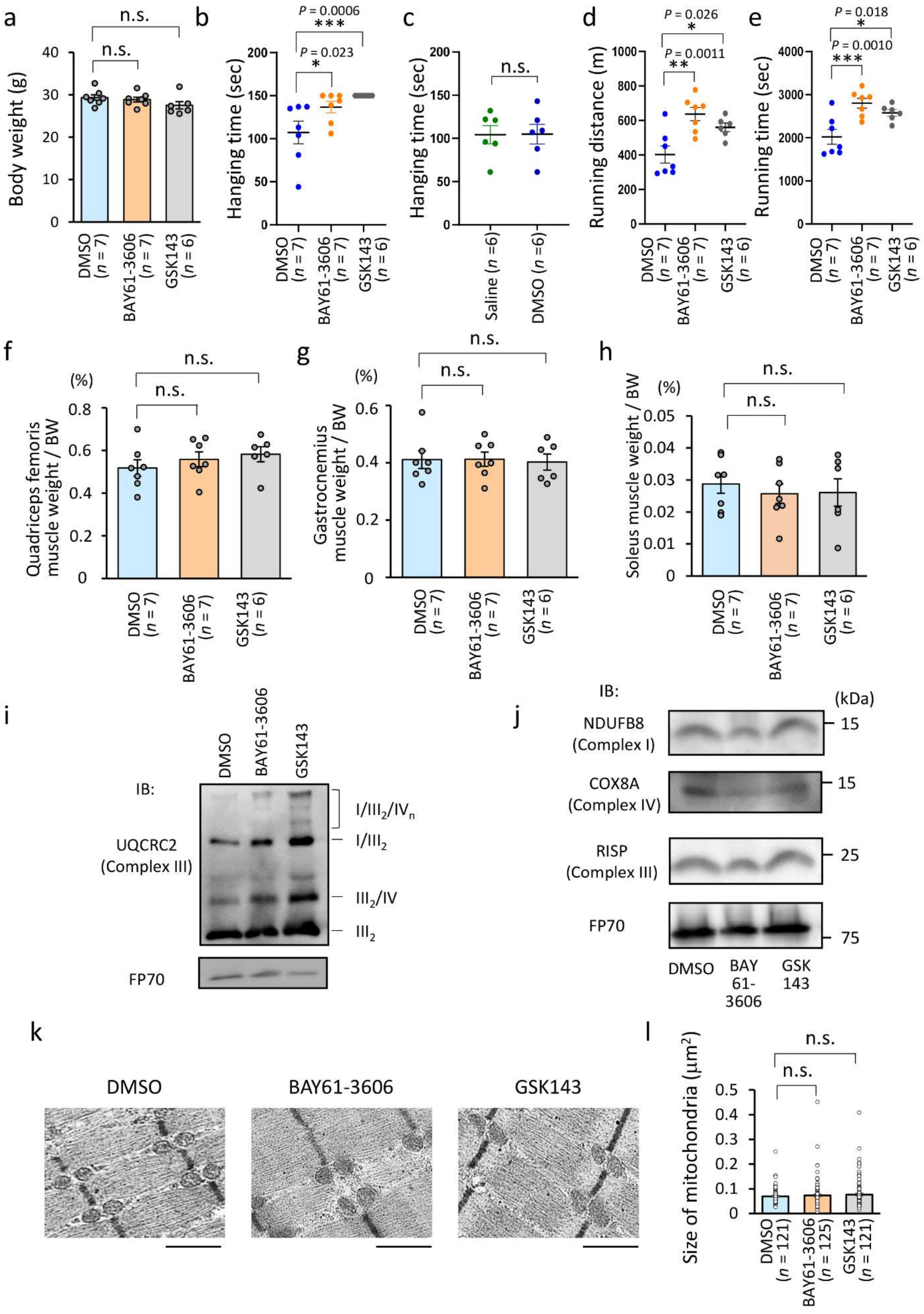
a
n.s.
n.s.
Body weight (g)
DMSO (n = 7)
BAY61-3606 (n = 7)
GSK143 (n = 6)
b
P = 0.0006
***
P = 0.023
*
Hanging time (sec)
c
n.s.
Saline (n =6)
DMSO (n =6)
d
P = 0.026
*
P = 0.0011
**
Running distance (m)
e
P = 0.018
*
P = 0.0010
***
Running time (sec)
f
Quadriceps femoris muscle weight / BW
(%)
g
Gastrocnemius muscle weight / BW
h
Soleus muscle weight / BW
i
IB:
UQCRC2 (Complex III)
FP70
I/III2/IVn
I/III2
III2/IV
III2
j
NDUFB8 (Complex I)
COX8A (Complex IV)
RISP (Complex III)
(kDa)
15
25
75
BAY 61-3606
GSK 143
k
l
Size of mitochondria (μm2)
DMSO (n = 121)
BAY61-3606 (n = 125)
GSK143 (n = 121)

Supplementary Fig. 6. Increased exercise performance in mice treated with SYK inhibitors. **a**, Body weight in mice injected with DMSO, BAY61-3606 (2 mg/kg) or GSK143 (2 mg/kg) for 6 weeks. Data are presented as means ± SE. n.s., not significant; two-sided Dunnett's test. **b**, Wire hanging time in mice injected with indicated reagents for 3 weeks. Data are presented as means ± SE. *P < 0.05; ***P < 0.001; Kruskal-Wallis test and post-hoc Mann-Whitney *U*-test. **c**, Wire hanging time in mice after 3-week injection of physiological saline or DMSO. Data are presented as means ± SE. n.s., not significant; unpaired two-sided Student's *t*-test. **d**, **e**, Results of forced treadmill exercise test after 4-week injection of indicated reagents. Data are presented as means ± SE. *P < 0.05; **P < 0.01; ***P < 0.001; two-sided Dunnett's test. **f-h,** Muscle weights of mice injected with indicated reagents for 6 weeks. Average weights of quadriceps femoris (**f**), gastrocnemius (**g**), or soleus (**h**) muscles from a pair of legs normalized to each body weight. Data are presented as means ± SE. n.s., not significant; two-sided Dunnett's test. Number of mice used in panels **a-h** is individually indicated. **i**, BN-PAGE for mitochondrial proteins of quadriceps femoris muscle from mice treated with indicated reagents. Positions corresponding to indicated mitochondrial supercomplexes and dimerized complex III (III_2) are indicated. Immunoblot (IB) was probed with anti-UQCRC2. FP70 was analyzed as an internal control. **j**, SDS-PAGE for mitochondrial proteins of quadriceps femoris muscle from mice treated with indicated reagents. IB was probed with antibodies against distinct respiratory complexes. For **i** and **j,** experiments were repeated twice and the results of one experiment are shown. **k**, Representative transmission electron micrographs of soleus muscle from mice after 6-week injection of indicated reagents. Scale bars; 1 μm. **l**, Mitochondria size in the soleus muscle from mice (n = 1, DMSO; n = 2, BAY61-3606; n = 2, GSK143) as panel k quantified by transmission electron microscopic examination of three sections each. Indicated number of mitochondria was evaluated. Data are presented as means ± SE. n.s., not significant; two-sided median test. Source data are provided as a Source Data file.

Supplementary Figure 3a

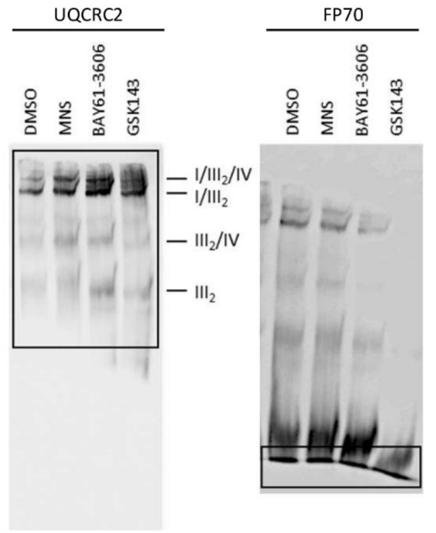

Supplementary Figure 3b

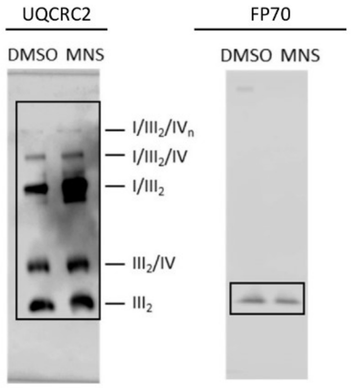

Supplementary Figure 3c

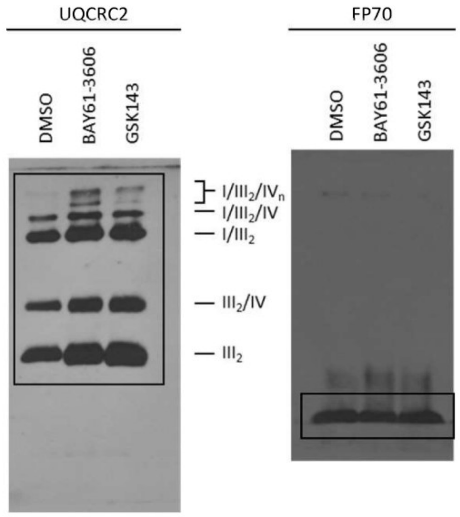

Supplementary Figure 3d

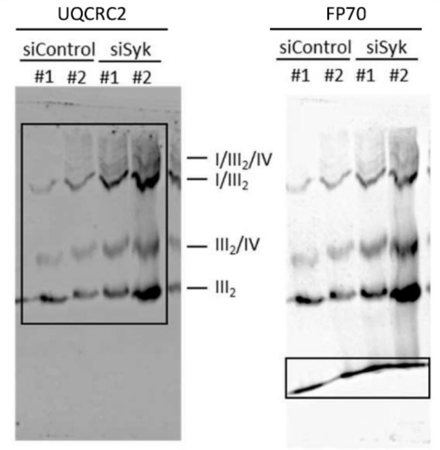

Supplementary Figure 3e

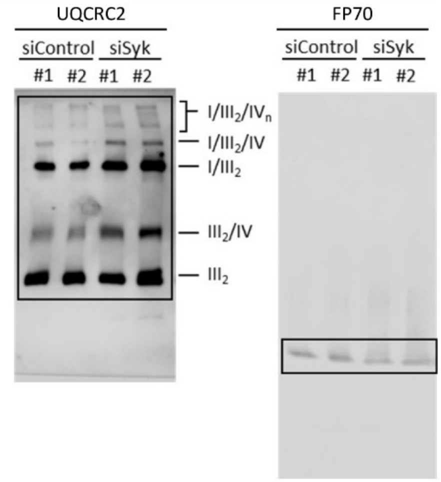

Supplementary Figure 6i

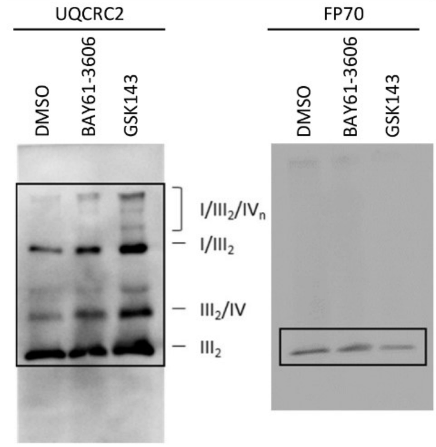

Supplementary Figure 6j

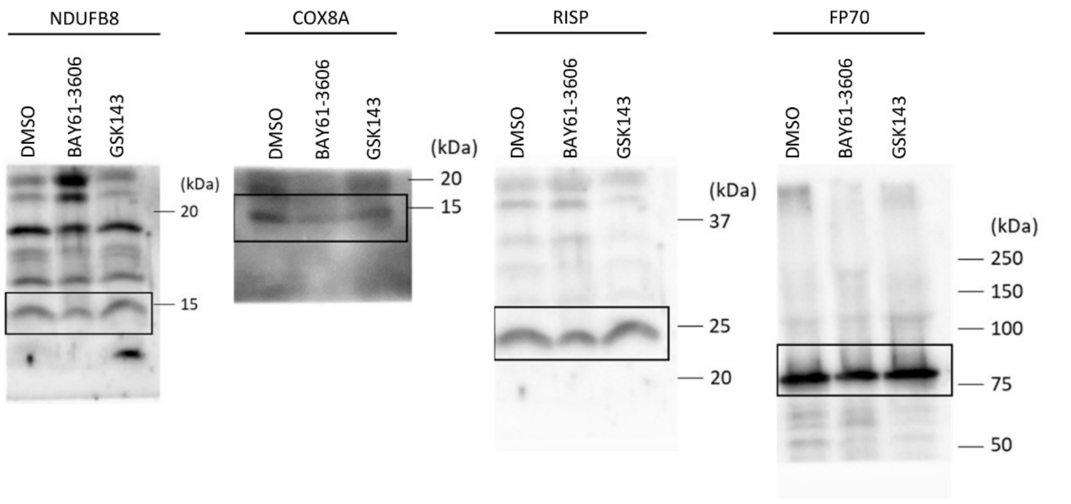

Supplementary Fig. 7. Uncropped scans of the immunoblots presented in Supplementary Fig. 3a-e and Supplementary Fig. 6i, j.

Supplementary Movie 1. A 30-second capture of DMSO- and MNS-treated mice subjected to a forced treadmill exercise, related with Fig. 6c, d. MNS-treated mice maintained the running pace whereas DMSO-treated mice could not.

Supplementary Movie 2. A 30-second capture of DMSO-, BAY61-3606-, and GSK143-treated mice subjected to a forced treadmill exercise, related with Supplementary Fig. 6d, e. BAY61-3606- and GSK143-treated mice maintained the running pace whereas DMSO-treated mice could not.
