# Supplementary material for: A FRET-based respirasome assembly screen identifies spleen tyrosine kinase as a target to improve muscle mitochondrial respiration and exercise performance in mice
Source: Nat Commun. 2023 Jan 25;14:312. doi: 10.1038/s41467-023-35865-x (PMC9877034; doi:10.1038/s41467-023-35865-x)
Supplement: Supplementary file 3 — Description of Additional Supplementary Files [file 41467_2023_35865_MOESM3_ESM.docx]

**Description of Additional Supplementary Files**

**Supplementary Movie 1:** A 30-second capture of DMSO- and MNS-treated mice subjected to a forced treadmill exercise, related with Fig. 6c, d. MNS-treated mice maintained the running pace whereas DMSO-treated mice could not.

**Supplementary Movie 2:** A 30-second capture of DMSO-, BAY61-3606-, and GSK143-treated mice subjected to a forced treadmill exercise, related with Supplementary Fig. 6d, e. BAY61-3606- and GSK143-treated mice maintained the running pace whereas DMSO-treated mice could not.

**Supplementary Data 1:** Results of FRET-based high-throughput screen with a library of 1280 compounds (LOPAC 1280)

**Supplementary Data 2:** Results of the secondary screen
