# Supplementary material for: A FRET-based respirasome assembly screen identifies spleen tyrosine kinase as a target to improve muscle mitochondrial respiration and exercise performance in mice
Source: Nat Commun. 2023 Jan 25;14:312. doi: 10.1038/s41467-023-35865-x (PMC9877034; doi:10.1038/s41467-023-35865-x)
Supplement: Supplementary file 8 — Reporting Summary [file 41467_2023_35865_MOESM8_ESM.pdf]

nature portfolio

Corresponding author(s): Satoshi Inoue

Last updated by author(s): Dec 6, 2022

Reporting Summary

Nature Portfolio wishes to improve the reproducibility of the work that we publish. This form provides structure for consistency and transparency in reporting. For further information on Nature Portfolio policies, see our Editorial Policies and the Editorial Policy Checklist.

Statistics

For all statistical analyses, confirm that the following items are present in the figure legend, table legend, main text, or Methods section.

n/a	Confirmed	
☐	☒	The exact sample size (*n*) for each experimental group/condition, given as a discrete number and unit of measurement
☐	☒	A statement on whether measurements were taken from distinct samples or whether the same sample was measured repeatedly
☐	☒	The statistical test(s) used AND whether they are one- or two-sided<br>*Only common tests should be described solely by name; describe more complex techniques in the Methods section.*
☒	☐	A description of all covariates tested
☐	☒	A description of any assumptions or corrections, such as tests of normality and adjustment for multiple comparisons
☐	☒	A full description of the statistical parameters including central tendency (e.g. means) or other basic estimates (e.g. regression coefficient) AND variation (e.g. standard deviation) or associated estimates of uncertainty (e.g. confidence intervals)
☐	☒	For null hypothesis testing, the test statistic (e.g. *F*, *t*, *r*) with confidence intervals, effect sizes, degrees of freedom and *P* value noted<br>*Give P values as exact values whenever suitable.*
☒	☐	For Bayesian analysis, information on the choice of priors and Markov chain Monte Carlo settings
☒	☐	For hierarchical and complex designs, identification of the appropriate level for tests and full reporting of outcomes
☒	☐	Estimates of effect sizes (e.g. Cohen's *d*, Pearson's *r*), indicating how they were calculated

Our web collection on statistics for biologists contains articles on many of the points above.

Software and code

Policy information about availability of computer code

Data collection	Fluorescent images of the cells expressing proteins fused with fluorophores were visualized with confocal fluorescence microscopy, Fluoview 10i (Olympus, Tokyo, Japan) or TCS SP8 (Leica Microsystems, Wetzlar, Germany) and images showing FRET efficiency were reconstructed using software attached to each fluorescence microscope (Fluoview 10i FRET package, FRET SE-Leica SP8). FRET signals in the cells co-expressing AcGFP and DsRed-Monomer in a 96-well plate were quantified using IN Cell Investigator image analysis software version 1.6.2 (GE Healthcare).
Data analysis	Statistical analyses were conducted using Excel Statistics 2010 (add-in software for Microsoft Excel) (SSRI, Tokyo, Japan), GraphPad Prism 8 (GraphPad Software, San Diego, CA, USA), or JMP version 9.0.0 (SAS Institute, Cary, NC, USA).

For manuscripts utilizing custom algorithms or software that are central to the research but not yet described in published literature, software must be made available to editors and reviewers. We strongly encourage code deposition in a community repository (e.g. GitHub). See the Nature Portfolio guidelines for submitting code & software for further information.

Data

Policy information about availability of data

All manuscripts must include a data availability statement. This statement should provide the following information, where applicable:

- Accession codes, unique identifiers, or web links for publicly available datasets
- A description of any restrictions on data availability
- For clinical datasets or third party data, please ensure that the statement adheres to our policy

Source data are provided with this paper. All the other data supporting the findings of this study are available from the corresponding authors upon reasonable request, as we have another project underway based on these data.

Human research participants

Policy information about studies involving human research participants and Sex and Gender in Research.

Reporting on sex and gender	No research involving human research participants was performed in the present study.
Population characteristics	No research involving human research participants was performed in the present study.
Recruitment	No research involving human research participants was performed in the present study.
Ethics oversight	No research involving human research participants was performed in the present study.

Note that full information on the approval of the study protocol must also be provided in the manuscript.

Field-specific reporting

Please select the one below that is the best fit for your research. If you are not sure, read the appropriate sections before making your selection.

☒ Life sciences ☐ Behavioural & social sciences ☐ Ecological, evolutionary & environmental sciences

For a reference copy of the document with all sections, see nature.com/documents/nr-reporting-summary-flat.pdf

Life sciences study design

All studies must disclose on these points even when the disclosure is negative.

Sample size	Sample-size of each experiments was described in figure legends. The in vitro sample size was completed according to enable statistical analyses. The sample-size of animal experiments was determined based on our previous work (Ikeda K. et al. Nat. Commun. 4, 2147, 2013) and recent papers (e.g. Janice Sánchez B. et al. Nat. Commun. 10, 4171, 2019; Roichman A, et al. Nat. Commun. 12, 3208, 2021). No sample-size calculation was performed.
Data exclusions	No data were excluded.
Replication	All attempts at replication were successful. The number of replication for each experiment is described in the figure legends.
Randomization	All allocation was random.
Blinding	The investigators were blinded to group allocation.

Reporting for specific materials, systems and methods

We require information from authors about some types of materials, experimental systems and methods used in many studies. Here, indicate whether each material, system or method listed is relevant to your study. If you are not sure if a list item applies to your research, read the appropriate section before selecting a response.

Materials & experimental systems

n/a	Involved in the study
☐	☒ Antibodies
☐	☒ Eukaryotic cell lines
☒	☐ Palaeontology and archaeology
☐	☒ Animals and other organisms
☒	☐ Clinical data
☒	☐ Dual use research of concern

Methods

n/a	Involved in the study
☒	☐ ChIP-seq
☒	☐ Flow cytometry
☒	☐ MRI-based neuroimaging

Antibodies

Antibodies used	Mouse monoclonal NDUFB8 antibody (ab110242, clone: 20E9DH10C12), rabbit monoclonal NDUFB8 antibody (ab192878, clone: EPR15961), mouse monoclonal RISP antibody (ab14746, clone: 5A5), rabbit monoclonal ATP5A antibody (ab176569, clone: EPR13030 (B)) and mouse monoclonal UQCRC2 antibody (ab14745, clone: 13G12AF12BB11) were purchased from Abcam (Cambridge, UK). Mouse monoclonal FP70 antibody (clone: 2E3GC12FB2AE2) was purchased from Invitrogen (Waltham, MA, USA). Rabbit polyclonal COX8A antibody (15368-1-AP) was purchased from Proteintech (Rosemont, IL, USA). Rabbit monoclonal SYK antibody (clone: D3Z1E) was obtained from Cell Signaling Technology (Beverly, MA, USA). Mouse monoclonal β-actin antibody (clone: AC-74) was purchased from Sigma (St. Louis, MO, USA). Rabbit polyclonal COX7RP antibody was raised in our laboratory (Ikeda, K. et al. Nat. Commun. 4, 2147, 2013). Horseradish peroxidase (HRP)-conjugated sheep anti-mouse immunoglobulin G (IgG) antibody (NA931V, lot: #17317435) and HRP-conjugated sheep anti-rabbit IgG antibody (NA934V, lot: #17320421) were purchased from GE Healthcare (Buckinghamshire, UK).
Validation	Regarding all antibodies, their application to western blot was validated by the companies (Abcam, Invitrogen, Proteintech, Cell Signaling Technology, Sigma, GE Healthcare) or validated in our previous study (Ikeda, K. et al. Nat. Commun. 4, 2147, 2013). All primary antibodies can detect mouse proteins.

Eukaryotic cell lines

Policy information about cell lines and Sex and Gender in Research

Cell line source(s)	The murine myoblastic cell line, C2C12, was obtained from ATCC (Manassas, VA, USA).
Authentication	Authentication of cell lines were done by STR analysis at the outside laboratory.
Mycoplasma contamination	All cell lines were negative for Mycoplasma contamination.
Commonly misidentified lines (See ICLAC register)	None

Animals and other research organisms

Policy information about studies involving animals; ARRIVE guidelines recommended for reporting animal research, and Sex and Gender in Research

Laboratory animals	Seven-week-old male DBA/2CrSlc mice were purchased from Japan SLC, Inc. (Shizuoka, Japan) and were maintained in a specific pathogen-free mouse facility of Tokyo Metropolitan Institute of Gerontology at a temperature of 22±2°C, a relative humidity of 55 ± 5%, and a 12:12 hour light:dark cycle (lights on, 08:00 to 20:00), with free access to water and Low Irradiated Diet (CRF-1, LID6, Oriental Yeast Co., Ltd., Tokyo, Japan).
Wild animals	None
Reporting on sex	Sex was not considered in the present study.
Field-collected samples	None
Ethics oversight	Animal care was in accordance with the Tokyo Metropolitan Institute of Gerontology animal experiment guidelines. The ethics committee of animal experiments at the Tokyo Metropolitan Institute of Gerontology approved our study protocol (approval no. 18021).

Note that full information on the approval of the study protocol must also be provided in the manuscript.
